# ING5 differentially regulates protein lysine acetylation and promotes p300 autoacetylation

**DOI:** 10.18632/oncotarget.22176

**Published:** 2017-10-31

**Authors:** Tao Zhang, Jin Meng, Xinli Liu, Xutao Zhang, Xiaojun Peng, Zhongyi Cheng, Feng Zhang

**Affiliations:** ^1^ Department of Thoracic Surgery, Tangdu Hospital, Fourth Military Medical University, Xi’an 710038, China; ^2^ Department of Pharmacology, Key Laboratory of Gastrointestinal Pharmacology of Chinese Materia Medica of the State Administration of Traditional Chinese Medicine, School of Pharmacy, Fourth Military Medical University, Xi’an 710032, China; ^3^ Department of Pharmacy, No. 309 Hospital of PLA, Beijing 100091, China; ^4^ Department of Bioinformatics, Jingjie PTM Biolab (Hangzhou) Co. Ltd, Hangzhou 310018, China

**Keywords:** inhibitor of growth 5 (ING5), quantitative proteomics, acetylation, p300 autoacetylation, lung cancer

## Abstract

ING5 belongs to the Inhibitor of Growth (ING) candidate tumor suppressor family. Previously, we have shown that ING5 inhibits invasiveness of lung cancer cells by downregulating EMT-inducing genes. However, the underlying mechanisms remain unclear. The aim of the study was to use integrated approach involving SILAC labeling and mass spectrometry-based quantitative proteomics to quantify dynamic changes of acetylation regulated by ING5 in lung cancer cells. Here, we have found that ING5 has a profound influence on protein lysine acetylation with 163 acetylation peptides on 122 proteins significantly upregulated and 100 acetylation peptides on 72 proteins downregulated by ING5 overexpression. Bioinfomatic analysis revealed that the acetylated proteins upregulated by ING5 located preferentially in nucleus to cytoplasm and were significantly enriched in transcription cofactor activity, chromatin binding and DNA binding functions; while those downregulated by ING5 located preferentially in cytoplasm rather than nucleus and were functionally enriched in metabolism, suggesting diverse functions of ING5 through differentially regulating protein acetylation. Interestingly, we found ING5 overexpression promotes p300 autoacetylation at K1555, K1558 and K1560 within p300 HAT domain, and two novel sites K1647 and K1794, leading to activation of p300 HAT activity, which was confirmed by accelerated acetylation of p300 target proteins, p53 at k382 and histone H3 at K18. A specific p300 HAT inhibitor C646 impaired ING5-increased acetylation of H3K18 and p53K382, and subsequent expression of p21 and Bax. In conclusion, our results reveal the lysine acetylome regulated by ING5 and provide new insights into mechanisms of ING5 in the regulation of gene expression, metabolism and other cellular functions.

## INTRODUCTION

Lysine acetylation is a reversible and precisely regulated posttranslational modification (PTM) which plays a key role in a wide range of cellular processes such as gene transcription, DNA binding, cell metabolism and signal transduction. Deregulation of protein acetylation has been associated with several diseases, especially cancer [1–3]. The acetylation of histones and non-histone proteins is catalyzed by histone acetyltransferases (HATs), which transfer the acetyl group from acetyl-CoA to the ε-amino group of a lysine residue. The reverse reaction is catalyzed by histone deacetylases (HDACs). A growing number of cellular proteins have now been identified to be acetylated by proteomic studies [4]. However, the functions and regulating mechanisms of this modification in diverse cellular proteins remain largely unknown.

The Inhibitor of Growth (ING) proteins (ING1-ING5) have been identified and characterized as candidate tumor suppressors and are implicated in the control of cell growth, senescence, apoptosis, DNA repair and chromatin remodeling [5–7]. All ING proteins contain a highly conserved carboxy-terminal plant homeodomain (PHD) finger which has been identified as binding motif to DNA, RNA and proteins, and found in nuclear proteins thought to be involved in chromatin mediated transcriptional regulation. In addition, the ING proteins also contain a unique amino-terminal regions which associate with distinct components of histone deacetylase (HDAC) and histone acetyltransferase (HAT) complexes. Evidence have shown that ING1 could associate with both HAT and HDAC protein complexes [8]; ING2 is part of the histone deacetylase complex Sin3A similar to ING1 [9]. ING3 is a component of the hNuA4 HAT complex which is responsible for acetylation of histone H4 and H2A in yeast [10]. ING4 associates with the HBO1 HAT, while ING5 associates with HBO1 and MOZ/MORF to form two distinct HAT complexes [11]. Thus, though ING proteins have no HAT activity, they regulate protein acetylation by association with different HATs and HDACs.

ING5 is the new member of ING family and diverse functions of ING5 have been reported since it was first identified by computational homology search. The pilot study of ING5 has shown that ING5 interacts with p300 and p53 in vivo, and its overexpression induces apoptosis in colorectal cancer cells [12]. In a yeast-two-hybrid screening of interaction partner for INCA1 which interacts with and inhibits cyclin A1/CDK2 activity [13], we identified ING5 as a co-factor of INCA1 which is indispensable for ING5's antiproliferative effects in MEF cells [14]. Recently, Mulder KW et al. [15] have identified ING5 as a component of genetic interacting network to control epidermal differentiation and protect epidermal stem cells from premature differentiation. These results indicate that ING5 plays an important role in the control of cell growth, apoptosis and differentiation. Recently, we have found that ING5 overexpression could inhibit migration and invasion of lung cancer cells by differentially regulating gene transcription, especially preventing expression of EMT-inducing genes [16]. However, the underlying mechanisms remain largely unknown. Given that ING5 acts as a cofactor of different HATs in regulating acetylation of histone and non-histone proteins [11], we postulate that ING5 may affect protein acetylome thus regulate gene transcription and protein function.

The purpose of this study was to define protein acetylation substrates and the lysine residues regulated by ING5 overexpression. By SILAC labeling and mass spectrometry-based quantitative proteomics, we have identified acetylome affected by ING5 overexpression, which reveal that ING5 differentially regulates lysine acetylation of proteins involved in DNA binding and metabolism. ING5 promotes p300 autoacetylation as well as acetylation of histone H3 and H4, while decreases acetylation of poteins involved in glycolysis and HIF-1 signaling pathway. Our results, for the first time, demonstrate that ING5 functions as a tumor suppressor by differentially and precisely regulating protein acetylation, in an indirect manner.

## RESULTS

### Lysine acetylation proteome in human NSCLC A549 cells

**Figure 1:**
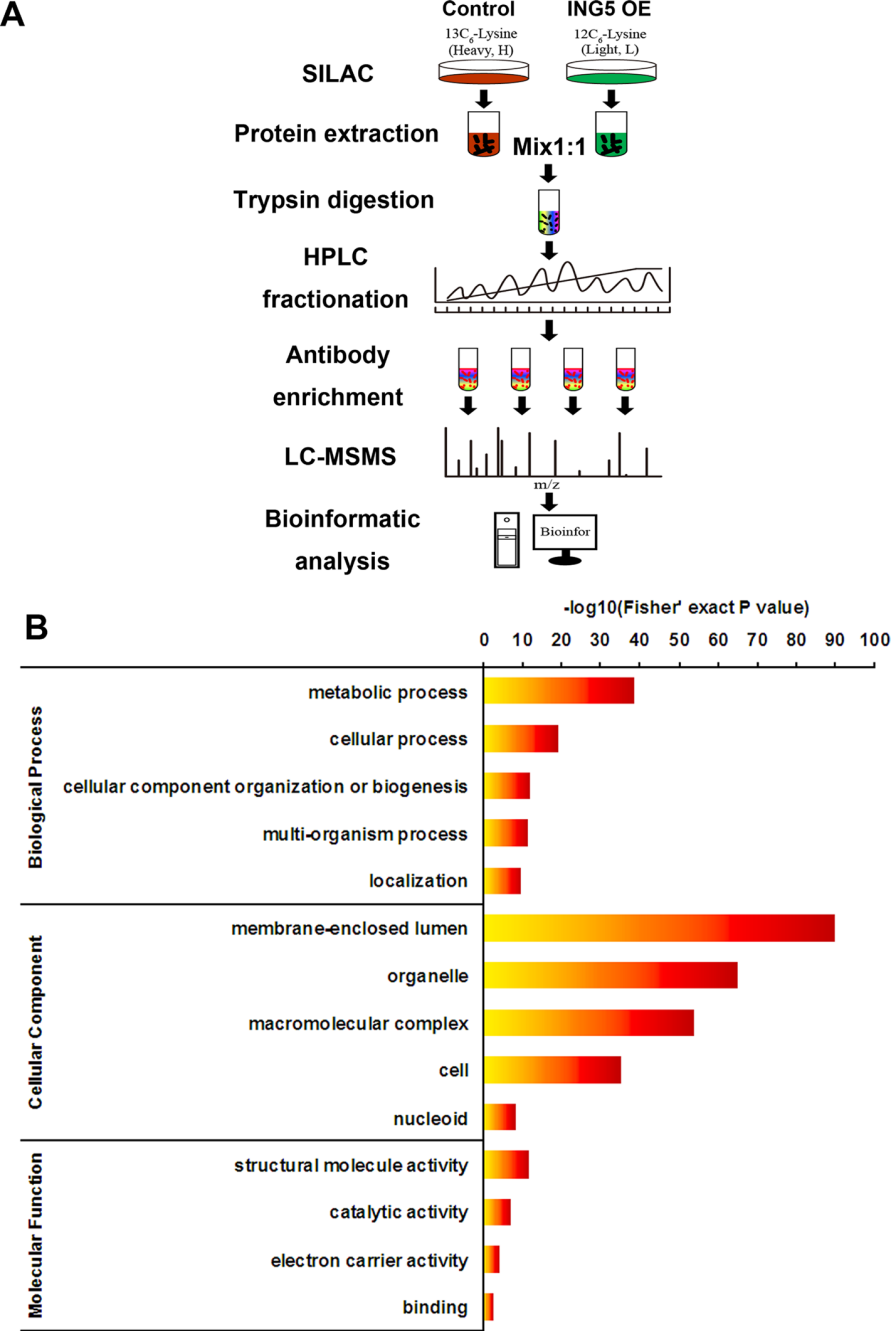
Profiling Lys acetylation proteome in control and ING5 overexpression lung cancer A549 cells (**A**) Schematic representation of experimental workflow for the SILAC quantification of Lys acetylation in control and ING5 overexpression (OE) A549 cells. (**B**) Gene Ontology (GO) analysis showing representative annotations of acetylated proteins.

To identify acetylated proteins and lysine sites influenced by ING5, we have generated stable ING5 overexpressing A549 cell line (A549 ING5) and control cell line (A549 control) described previously [16] and quantified the acetelomes between two cell lines using stable isotope labeling of amino acids in cell culture (SILAC) and MS (Figure 1A). In total, 1291 acetylation peptides from 648 proteins were identified, and 1222 acetylation peptides on 626 proteins were quantifiable. An excel file containing the information of acetylation peptides and proteins is enclosed in Supplementary Table 1. Some proteins were highly acetylated in A549 cells, including plectin with 20 sites, DNA-dependent protein kinase catalytic subunit (PRKDC) with 18 sites and retinal dehydrogenase 1 (ALDH1A1) with 13 sites.

### Gene ontology annotation proteome of lysine acetylation

Consistent with previous reports [2], our data show that Lys-acetylated proteins are significantly enriched in biological processes including metabolic process, cellular process, cellular component organization or biogenesis, multi-organism process and localization (Figure 1B). The most enriched Gene Ontology terms of cellular component include membrane-enclosed lumen, organelle, macromolecular complex, cell and nucleoid (Figure 1B). The molecular functions of Lys-acetylated proteins include structural molecule activity, catalytic activity, electron carrier activity and binding (Figure 1B).

### Protein interaction networks and protein complexes of Lys acetylation proteome

We analyzed the protein-protein interaction networks of Lys-acetylated proteins on the basis of the STRING database. Our data show a widely connected network with clusters of nodes to form protein complex patterns (Supplementary Figure 1). Using the MCODE tool, we have identified a number of highly connected subnetworks among Lys-acetylated proteins, including ribosome and spliceosome, PA700-20S-PA28 complex, CCT micro-complex and Arp2/3 protein complex (Supplementary Figure 2), suggesting a potential role of ING5 on regulating the function of these complexes by affecting protein lysine acetylation.

### Quantification of protein acetylation and Lys sites in response to ING5 overexpression

**Figure 2:**
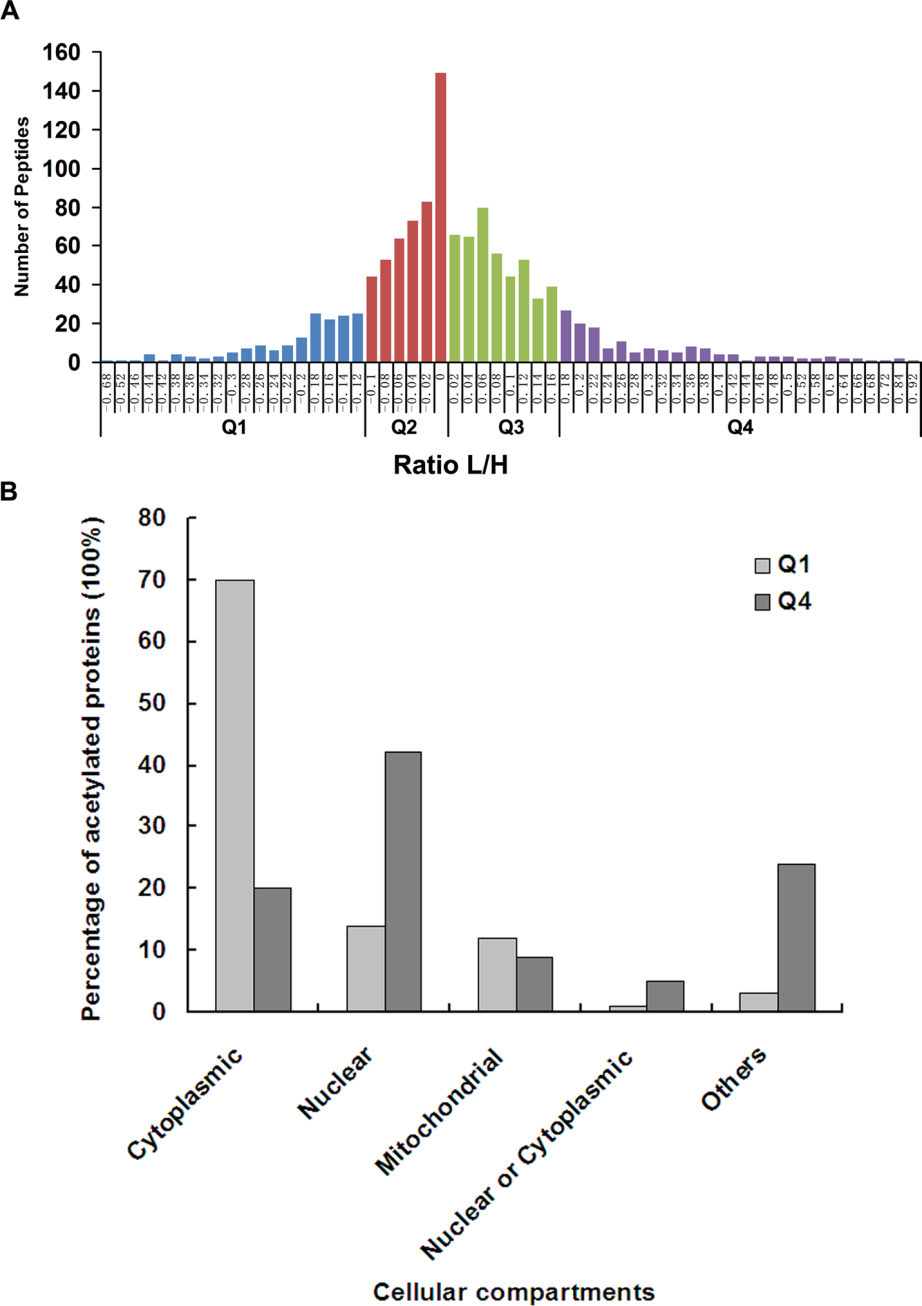
Quantitative analysis of lysine acetylation proteome upon ING5 overexpression (**A**) Quantitative ratio (L/H) distribution of acetylated protein influenced by ING5 overexpression. A549 control cells were labeled with 13C-Lysine (Heavy, H) and A549 ING5 cells were labeled with 12C-Lysine (Light, L). The figure shows the protein ratio [log (protein ratio), with base=2] of each differentially expressed protein. Proteins with upregulated acetylation are located right of zero of X-axis, while proteins with downregulated acetylation are located left of zero of X-axis. (**B**) Subcellular localization of lys-acetylated proteins regulated by ING5 overexpression. Cellular distributions of proteins in Q1 and Q4 are shown according to subcellular localization annotation.

Using SILAC, we have quantified the dynamics of Lys acetylation sites in control and ING5 overexpressing A549 cells. Quantified acetylated proteins in this study were divided into four quantiles. The average (x) and standard deviation (y) of the Log2L/H SILAC ratios of all quantified acetylation peptides was calculated. The L/H SILAC ratio of each acetylation peptide was then transformed to a z-score based on z = (Log2Ratio – x)/y, where the ratio is the L/H SILAC ratio. The cutoff z-scores were set according to cumulative density function of normal distribution at three different percentiles – 15%, 50% and 85%. Each acetyaltion peptide was then allocated to the quantiles based on the transformed z-score. In this way, we generated four quantiles: Q1 (0~15%), Q2 (15~50%), Q3 (50~85%) and Q4 (85~100%) (Figure 2A). Our results show that ING5 overexpression has a profound influence on protein lysine acetylation. The overall acetylation level was normally distributed. Acetylation of proteins in Q4 (183 Ac-peptides) and deacetylation of proteins in Q1 (184 Ac-peptides) are considered to be promoted by ING5. Altogether, 100 acetylation peptides on 72 acetylation proteins were significantly downregulated (> 1.5 fold decrease) and 163 acetylation peptides on 122 acetylation proteins were significantly upregulated (> 1.5 fold increase) by ING5 overexpression in A549 cells. Though the amounts of Ac-peptide in Q4 and Q1 are almost equal, 24 or 66 showed over 3-fold or 2-fold increase in abundance, while only 2 or 25 showed over 3-fold or 2-fold decrease in ING5 overexpressing A549 cells.

### Cellular localization of the Lys acetylation Proteome regulated by ING5

We performed cellular compartment analysis of all acetylated proteins in A549 cells and differentially acetylated proteins regulated by ING5. The current study found Lysine acetylated proteins localized to distinct cellular compartments including cytoplasm (42%), nucleus (22%), mitochondria (15%), extracellular space (5.7%) and endoplasmic reticulum (ER) (4%). By comparing acetylome compartments of ING5 overexpressed A549 cells with that of control cells, we found that the acetylated proteins promoted by ING5 locates preferentially in nucleus (41%) rather than cytoplasm (20%). In contrast, the acetylated proteins decreased by ING5 locates preferentially in cytoplasm (70%) rather than nucleus (14%) (Figure 2B). Thus, ING5 may affect protein function through up or down regulating protein acetylation which affects protein cellular localization.

### Functional annotation of the lys acetylome regulated by ING5

**Figure 3:**
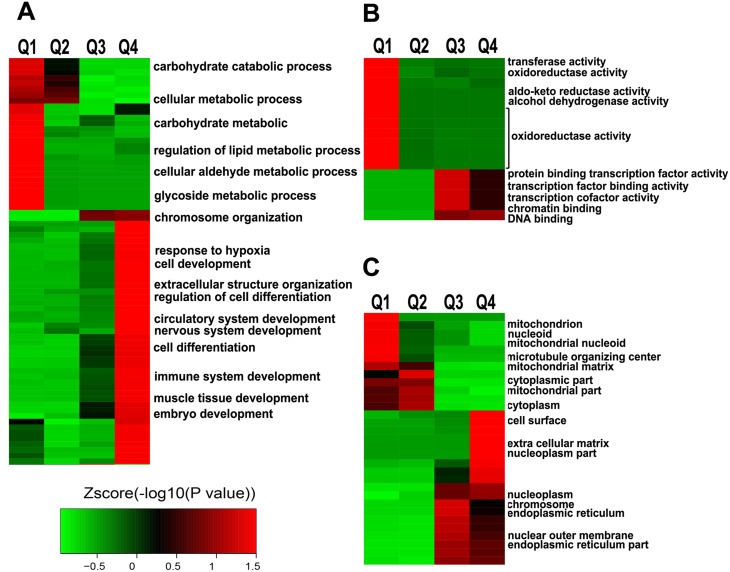
Enrichment and clustering analysis of Lys acetylation proteome regulated by ING5 based on Gene Ontology annotation (**A**) Biological process. (**B**) Molecular function. (**C**) Cellular component.

To understand the biological functions of Lys acetylation regulated by ING5, we performed enrichment analysis with the Gene Ontology annotation database. Our data show that ING5-promoted acetylated proteins are significantly enriched in biological processes including response to hypoxia, cell development, cell differentiation, immune system development, and so on; while ING5-downregulated acetylated proteins are enriched in various types of metabolic processes (Figure 3A, Supplementary Figure 3A). Analysis of molecular function show that acetylated proteins increased by ING5 are enriched in transcription factor binding activity, transcription cofactor activity, chromatin binding and DNA binding functions, suggesting that ING5 may regulate gene transcription via increasing protein acetylation (Figure 3B, Supplementary Figure 3B). In contrast, decreased acetylated proteins are enriched mainly in metabolic functions including transferase activity, oxidoreductase activity, aldo-keto reductase (NADP) activity and alcohol dehydrogenase (NADP+) activity (Figure 3B, Supplementary Figure 3B). Compartment analysis showed that ING5 has different effects on lysine acetylation of proteins distributed in different cellular compartment (Figure 3C, Supplementary Figure 3C).

**Figure 4:**
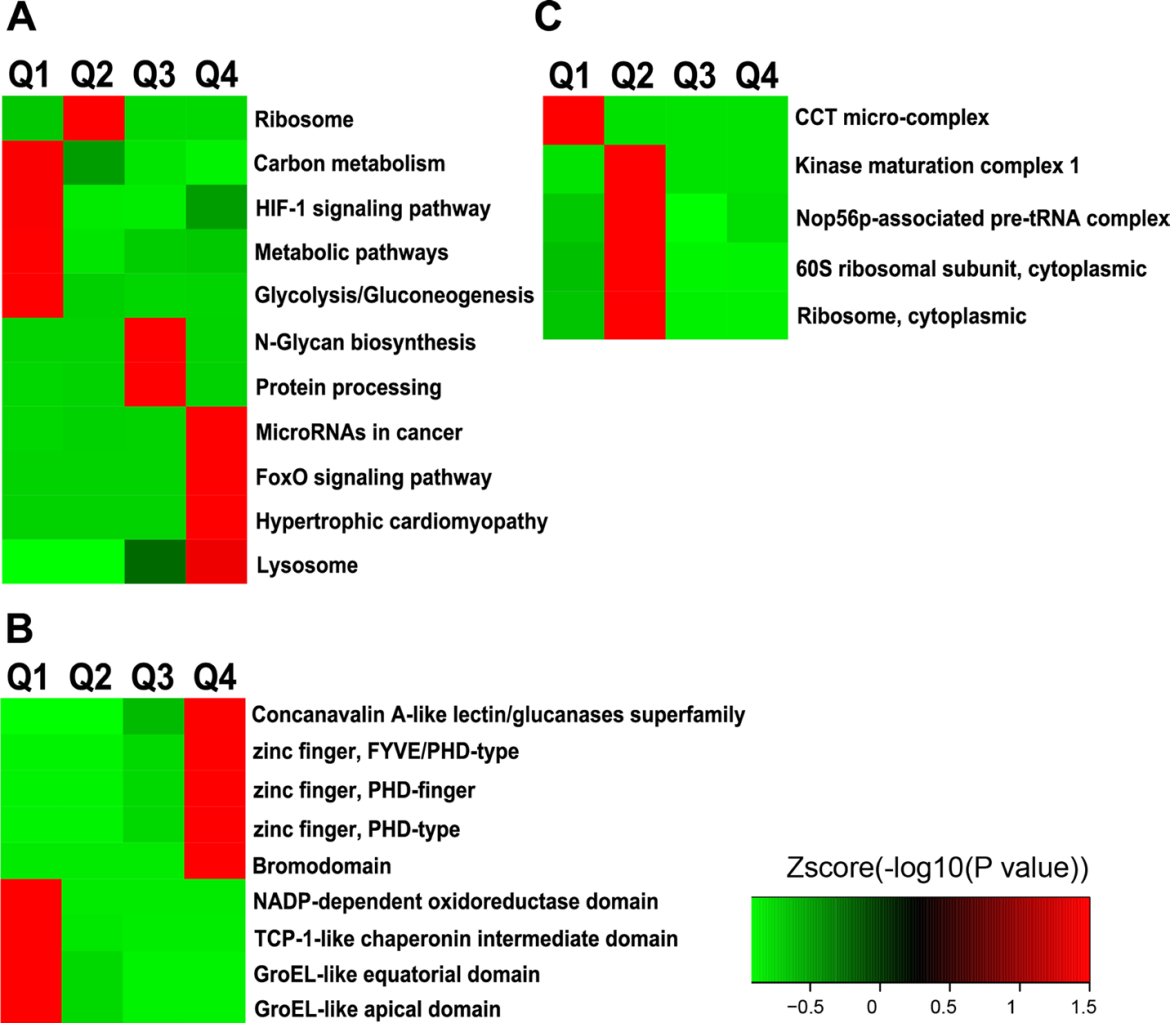
Enrichment and clustering analysis of Lys acetylation proteome regulated by ING5 based on KEGG pathways, protein domains and complexes (**A**) KEGG pathway analysis. (**B**) PFAM domain analysis. (**C**) CORUM protein complex analysis.

To understand the cellular pathways involved in ING5-regulated Lys acetylation, we performed enrichment analysis of KEGG pathways. Our data show that ING5 upregulated acetylated proteins are enriched in hypertrophic cardiomyopathy (*P* = 0.036), FoxO signaling pathway (*P* = 0.036), MicroRNAs in cancer (*P* = 0.050) and Lysosome (*P* = 0.050) (Figure 4A). In comparison, ING5-downregulated acetylated proteins are enriched in metabolic pathway (*P* = 0.001), glycolysis/gluconeogenesis (*P* = 0.001), HIF-1 signaling pathway (*P* = 0.043) and carbon metabolism (*P* = 0.049) (Figure 4A), suggesting that ING5 has diverse effects on cellular pathways by differentially regulating protein acetylation.

### Domain analysis and protein complex analysis by ING5 overexpression

ING5-increased acetylated-lysine peptides are enriched in domains of Zinc finger (FYVE/PHD-type, PHD-type and PHD-finger) (*P* = 0.030) and Bromodomain (*P* = 0.044) (Figure 4B). ING5-downregulated acetylated-lysine peptides are enriched in domains including TCP-1-like chaperonin intermediate domain (*P* = 0.013), GroEL-like apical or equatorial domain (*P* = 0.022) and NADP-dependent oxidoreductase domain (*P* = 0.028) (Figure 4B). These results suggest that ING5 may precisely regulate diverse cellular functions by up/down-regulating protein lysine acetylation.

To identify complexes regulated by ING5 and Lys acetylation, we performed protein complex enrichment analysis with Comprehensive Resource of Mammalian protein complexes (CORUM) database. We have identified significant enrichment of decreased acetylation in the CCT micro-complex (*P* = 0.005) by ING5 overexpression (Figure 4C). The SNF2h-cohesin-NuRD complex, which is implicated in chromatin remodeling, has been identified to be enriched with increased acetylation by ING5 overexpression with marginal significance (*P* = 0.052).

### ING5 overexpression affects acetylation of histone proteins

**Figure 5:**
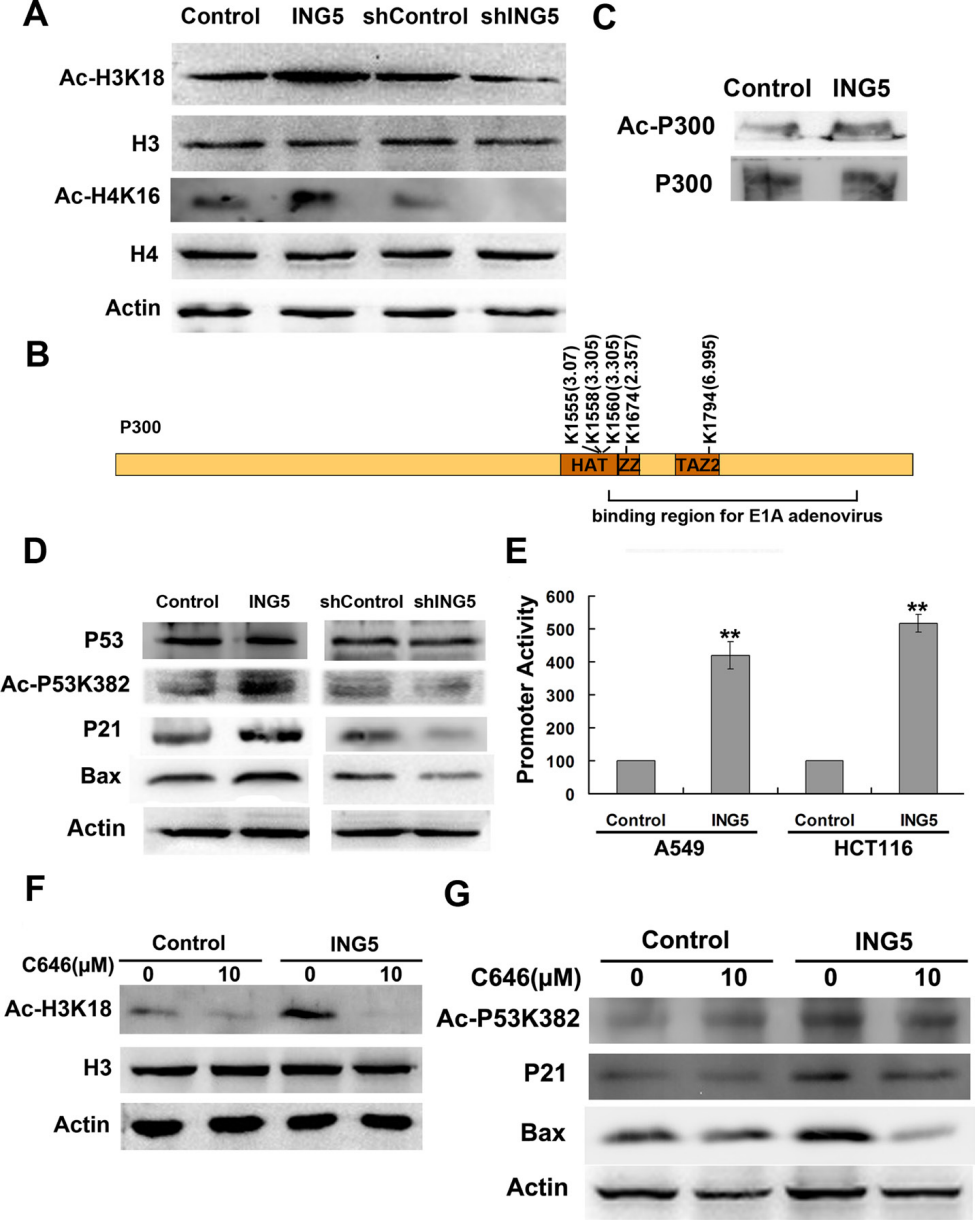
ING5 overexpression promotes histone acetylation and p300 autoacetylation (**A**) ING5 overexpression increases acetylation of Histone H3K18 and H4K16. (**B**) ING5 overexpression increases acetylated lysine sites of p300 by quantitative proteomics. (**C**) ING5 overexpression increases acetylation of p300 by IP-western. (**D**) ING5 overexpression promotes p53 acetylation at K382 and expression of its targets p21 and Bax. (**E**) ING5 overexpression increases p53-responsive promoter. Data are shown as mean plus standard error of three independent experiments. ^**^p < 0.01 compared to corresponding control. (**F**) P300 acetyltransferase (HAT) inhibitor C646 impairs ING5-promoted acetylation of H3K18. (**G**) C646 impairs ING5-promoted acetylation of p53K382 and expression of p53 targets p21 and Bax.

Enhanced histone acetylation has been regarded as a condition of transcriptionally active genes. Our data show that ING5 overexpression promotes acetylation of histone H3 at K18 and K23 by 1.9 fold, histone H4 at K16 and K12 by 3.4 fold and K8 by 3.1 fold. Histone H2B acetylation is also upregulated by ING5 to an even greater extent than H3 and H4 with K17 and K21 increased by 4.61 fold in H2B 2F, and K6 and K12 by 3.95 fold in H2B 1H. However, ING5 overexpression induces a decrease of Histone H1c K75 acetylation by 2.31 fold. Increased acetylation of H3K18 and H4K16 was confirmed by western blot in ING5-overexpressing A549 cells (Figure 5A). As a confirmation, in ING5-knockdown A549 cells, acetylation of H3K18 and H4K16 was decresed (Figure 5A).

### ING5 overexpression promotes p300 autoacetylation

Histone acetyltransferase p300 (EP300) functions as a transcriptional co-activator which is involved in regulation of a variety of genes. P300 could directly interact with many proteins including ING5. In the current study, ING5 increases acetylated level of p300 at K1555 (by 3.07 fold), K1558 and K1560 (by 3.305 fold) (Figure 5B). These lysine residues have been reported to be autoacetylated, suggesting that ING5 could promote autoacetylation of p300. We also found 2 novel acetylated lysine sites significantly upregulated by ING5, including K1794 (by 6.995 fold) and K1674 (by 2.357 fold) (Figure 5B). ING5-stimulated p300 acetylation was confirmed by IP-western (Figure 5C).

Western blot confirmed increased acetylation of histone H3K18 (Figure 5A) and p53 lys382 (Figure 5D), the known targets for p300 HAT, whose acetylation is decreased in ING5 knockdown cells (Figure 5A, 5D). ING5 increases acetylation of p53 K382 which in turn activates transactivation activity of p53. We have previously showed the activation of p53-responsive promoter by ING5 in MEF cells [14]. Using the same methods, we confirmed that ING5 overexpression increased activity of p53-responsive promoter in lung cancer A549 cells (Figure 5E). We also detected increased protein level of p53 target genes p21 and Bax, which were downregulated by ING5 knockdown (Figure 5D).

C646, a specific p300 acetyltransferase inhibitor, significantly impaired the promoting effects of ING5 overexpression on acetylation of p300 target proteins H3K18 and p53K382 (Figure 5F, 5G). In addition, C646 abrogated ING5 overexpression mediated upregulation of p53 targets p21 and Bax (Figure 5G). These results further confirmed that ING5 overexpression increased p300 autoacetylation and subsequent acetylation of its target proteins.

## DISCUSSION

### ING5 specifically and diversely regulates protein acetylation

ING5 has been identified as a component of HBO1 and MOZ/MORF HAT complexes [11] and also associates with p300 HAT [12], thus may participate in regulation of protein acetylation, chromatin remodeling and gene expression. The current study of quantitative acetylome represents the first systematic analysis of Lys-acetylated proteins in response to overexpression of ING5. We have identified 1291 acetylation peptides from 648 proteins and quantified acetylation changes of 1,222 sites in response to ING5 overexpression. The quantitative analysis shows that ING5 regulates protein lysine acetylation in dual directions. Altogether, 163 acetylation peptides on 122 proteins were significantly upregulated and 100 acetylation peptides on 72 proteins were significantly downregulated by ING5 overexpression.

The acetylated proteins promoted by ING5 locate preferentially in nucleus (42%) rather than cytoplasm (20%) and are functionally enriched in DNA binding. In contrast, the hypo-acetylated proteins induced by ING5 overexpression locate preferentially in cytoplasm (70%) rather than nucleus (14%) and are significantly enriched in metabolism. These results suggest that ING5 has diverse functions by differentially regulating protein acetylation.

### ING5 may regulate gene transcription by affecting acetylation level of all four histones and proteins involved in histone modification complexes

Post-translational modification of histone proteins in chromatin architecture plays a crucial role in the epigenetic regulation of gene transcription by consititution of “histone code”. Our data show that ING5 overexpression promotes acetylation of histone H2B, H3 and H4, whereas downregulates histone H1c acetylation, suggesting a complex regulatory effects of ING5 on gene transcription by altering histone acetylation status.

Doyon et al. [11] have reported that ING5 is present in two HAT complexes, with HBO1 HAT for acetylation of H4K5, H4K8 and H4K12, and with MOZ/MORF HAT for H3K14 acetylation. In consistent, our data show that ING5 overexpression promotes acetylation of H4 at K12 by 3.4 fold and K8 by 3.1 fold. In addition, we have detected increased acetylation of H3K18 and H4K16 by both acetylome and western blot in ING5 overexpression A549 cells, which might be an indirect effect of ING5 overexpression through influencing other HATs, as H3K18 is also a target of P300 [17], while H4K16 is a target of Tip60 [10].

Histone acetylation and methylation are two major modifications implicated in gene transcription regulation in response to various cellular signals. ING5, as a subunit of HBO1 and MOZ/MORF complexes [11], not only regulates acetylation of histone proteins, but also proteins involved in different complexes of chromatin remodeling. Choudhary et al. [4] have already found extensive acetylation of proteins that belong to different complexes involved in chromatin remodeling. The current data show that ING5 could promote lysine acetylation of proteins of HBO1 complex, including protein Jade 3 (PHF16) at K30 and K32 (by 2.37 fold) and protein Jade 2 (PHF15) at K298 (by 1.90 fold), but decreased the acetylation level of chromatin modification-related protein MEAF6 (by 1.85 fold). ING5 could also increase acetylation of proteins of MOZ/MORF complex, including bromodomain and PHD finger-containing protein 3 (BRPF3) at K546 (by 2.13) and K105 (by 1.92), but decrease bromodomain-containing protein 1 (BRD1) at K23 (by 1.82 fold). In addition, ING5 regulates lysine acetylation of proteins which are components of methyltransferase complex and HDAC complex. MLL3 (histone-lysine N-methyltransferase 2C, KMT2c) belongs to the mixed lineage leukemia (MLL) histone methyltransferases (HMTs) family which maintains active chromatin structure by methylating histone H3 at lysine 4 (H3K4) [18]. ING5 promotes MLL3 acetylation at two novel sites K2809 and K2814 (by 1.72 folds). Methyl-CpG-binding protein 2 (MeCP2) is a member of the methyl-cytosine-guanine (CG)-binding domain (MBD) family of proteins, which binds to Sin3A-HDAC complex and mediates transcription repression [19]. ING5 increases acetylation of MeCP2 at lys42 (by 1.431 folds), and HDAC1 at K100 (by 1.724 folds). Though the implications of the lysine acetylation of the proteins are not clear, these results suggest that ING5 may affect histone methylation and acetylation/deacetylation by regulating protein acetylation in different chromatin modification complexes thus have a widely impact on gene transcription regulation.

### ING5 overexpression promotes p300 autoacetylation and HAT activity

Histone acetyltransferase p300 functions as a transcriptional co-activator by forming complexes with a variety of transcription factors and remodeling chromatin through acetylating all four core histones (H2A, H2B, H3 and H4), thus giving an epigenetic tag for transcriptional activation [20, 21]. It also acetylates non-histone targets and plays a critical role in regulation of protein functions in various cellular processes.

ING5 was first identified to physically interact with both p53 and p300 and enhance acetylation of p53 at K382 by p300, thus stimulates the transactivation activity of p53 [12]. However, how ING5 regulates p300 functions was unknown. In the current study, we have found increased acetylation at 5 lysine sites of p300 by ING5 overexpression, among which K1555, K1558 and K1560 locate in the activation loop motif within the HAT domain, while K1674 and K1794 locate in the binding region of p300 for E1A adenovirus. Autoacetylation of several key lysine sites (K1499, K1549, K1554, K1558 and K1560) within the p300 HAT domain has been identified to stimulate p300 HAT activation. Our data suggest that ING5 activates p300 HAT by promoting p300 autoacetylation.

The increased p300 HAT activity upon ING5 overexpression was validated by upregulated acetylation of p300 target p53K382, which could in turn facilitate the sequence-specific DNA binding activity of p53 [22]. Consistently, we have observed an increased p53-responsive promoter activity, which is accompanied by higher protein level of p53 target genes p21 and Bax. As reviewed by Wang et al. [23], p300 functions as a co-activator through the following mechanisms: p300 bridges DNA-bound transcription factors and basal transcription machinery; p300 acetylates histones resulting in chromatin remodeling and relaxation of chromatin structure to enable transcription; p300 acetylates p53 C-terminal tail to increase its DNA binding activity. In the current study, we demonstrate that ING5 positively regulates p53 transactivation activity by increasing p300 HAT activity to acetylate both histones and p53. Pretreatment with a specific p300 HAT inhibitor C646 significantly impaired the promoting effects of ING5 on acetylation of p53K382 and abrogated ING5-induced upregulation of p53 targets p21 and Bax. These results suggest that ING5 is an important regulator of p300 HAT activity which plays an important role in ING5 functions. There are still lots of questions need to be elucidated, for example, how ING5 regulates the recruitment of p300, and what's the association between the mechanisms for p300-dependent p53 transactivation.

Increased H3K18 acetylation by ING5 overexpression was also investigated in the current quantitative acetylome data and verified by western blot, which was abrogated by p300 HAT inhibitor C646. These results further confirm that ING5 overexpression increases p300 autoacetylation and subsequent HAT activity. H3K18 is a preferential acetylation target of p300, and deletion of p300 dramatically reduces H3K18 acetylation [17]. Global H3K18 hypoacetylation strongly correlates with a more aggressive cancer phenotype and a poor prognosis in prostate, lung and kidney cancer patients [24–26]. ING5 increased H3K18 acetylation supports what we have previous reported that higher nuclear ING5 level predicts a better prognosis in lung cancer patients [16]. However, more clinical investigation is needed to explore the relating mechanisms.

Apart from the two typical p300 targets, transcription factor p53 and histone H3, Src substrate cortactin (CTTN), an oncogene implicated in cancer metastasis, is a newly found substrate of p300 for acetylation [27]. Interestingly, the current data reveal that ING5 overexpression increases acetylation of cortactin at K161 by 2.38 fold, K309 by 2.44 fold and K272 by 2.73 fold. Deacetylation of cortactin by SIRT1 [27] and HDAC6 [28] promotes cancer cell migration and invasion, which also could be observed in p300-null cells [29]. Whether p300-mediated cortactin acetylation promoted by ING5 is involved in ING5-suppressed invasiveness of lung cancer cells deserves further validation.

The role of p300 in tumorigenesis and progression is controversial. As p300 participates in transcriptional regulation of a variety of genes, including both tumor suppressor genes and genes related to cancer aggressiveness. Our results provide mechanistic insights into suppressed invasiveness of lung cancer cells by ING5 overexpression, where ING5-promoted p300 autoacetylation and HAT activity plays an important role. The mechanisms by which ING5 increases p300 autoacetylation and the effects of ING5 on p300 chromatin redistribution are under further investigation.

In summary, the current data suggest that ING5 functions as a tumor suppressor by diversely regulating protein lysine acetylation and provide new insights into mechanisms of ING5 inhibition of cancer invasiveness. Future investigation concerning the interaction of ING5 with different HAT or HDAC complexes, the confirmation and biological significance of specific lysine acetylation regulated by ING5 would further reveal the complex regulating network of ING5.

## MATERIALS AND METHODS

### Cell culture

Human lung cancer A549 cell line was purchased from the Type Culture Collection of the Chinese Academy of Sciences, Shanghai, China. A549 cells overexpressing ING5 (A549 ING5) and A549 control cells [14] were grown in Dulbecco's modified Eagle's medium (DMEM, Gibco, USA) supplemented with 10% fetal bovine serum (HyClone, USA), 10 mg/ml antibiotics (penicillin and streptomycin) and 2 mmol/L L-glutamine at 37°C under 5% CO_2_ and saturated moisture.

### SILAC labeling and cell harvest

A549 Control and A549 ING5 cells were maintained in SILAC media with ^13^C_6_-lysine (heavy) and ^12^C_6_-lysine (light) respectively over six doublings to reach ≈ 97% labeling efficiency. The cells were further expanded in SILAC media to desired cell number (~5 × 10^8^) in 30 × 150 cm^2^ flasks. When the cells were maintained in SILAC media for 80% confluence, they were harvested and washed twice with ice-cold PBS supplemented with 2 μM Trichostatin A and 30 mM Nicotinamide. After snap freezing in liquid nitrogen, cell pellets were stored in –80°C freezer for future use.

### Protein extraction

The harvested “heavy” and “light” labeled cells were lysed with 2×NETN buffer (200 mM NaCl, 100 mM Tris-Cl, 2 mM EDTA, 1.0%NP-40, pH7.2) supplemented with 0.5% Triton X-100 on ice for 30 min, respectively. The supernatants were saved after centrifuge at 20,000 g for 10 min at 4°C. After concentration measurement, equal amounts of crude proteins in supernatant labeled “heavy (H)” or “light (L)” were mixed and the crude proteins were precipitated by adding TFA with 15% final concentration (v/v) (soluble fraction). After washing twice with −20°C acetone, the proteins pellets were dissolved in 100 mM NH_4_HCO_3_ (pH 8.0) for trypsin digestion. Remaining cell pellets were dissolved in 8 M urea to extract the chromatin-binding proteins. Then, equal amount of chromatin-binding proteins in urea solution were mixed and the proteins were precipitated by adding TFA with 15% final concentration (v/v) (nuclear pellet fraction). After washing twice with −20°C acetone, the protein pellets were dissolved in 100 mM NH_4_HCO_3_ for trypsin digestion.

### Affinity enrichment

Trypsin (Promega) was added into protein solution for digestion. To enrich protein acetylation, tryptic peptides dissolved in NETN buffer (100 mM NaCl, 1 mM EDTA, 50 mM Tris-HCl, 0.5%NP-40, pH8.0) were incubated with pre-washed antibody beads (PTM Biolabs) at 4°C overnight with gentle shaking. The beads were washed four times with NETN buffer and twice with ddH_2_O. The bound peptides were eluted from the beads with 0.1% TFA. The eluted peptides were collected and vacuum-dried followed by analyzing by LC-MS/MS.

### LC-MS/MS analysis

Peptides were dissolved in 0.1% FA, and peptide separation was performed using a reversed-phase analytical column (Acclaim PepMap RSLC, Thermo Scientific) with a linear gradient of 5–35% solvent B (0.1% FA in 98% ACN) for 30 min and 35–80% solvent B for 10 min at a constant flow rate of 300 nl/min on an EASY-nLC 1000 UPLC system, The resulting peptides were analyzed by Q Exactive^TM^ Plus hybrid quadrupole-Orbitrap mass spectrometer (ThermoFisher Scientific). The peptides were subjected to NSI source followed by tandem mass spectrometry (MS/MS) in Q Exactive^TM^ Plus (Thermo) coupled online to the UPLC. For MS scans, the m/z scan range was 350 to 1600Da.

### Database search

The resulting MS/MS data were processed using MaxQuant with integrated Andromeda search engine (v.1.4.1.2). Tandem mass spectra were searched against SwissProt_human database concatenated with reverse decoy database. Trypsin/P was specified as cleavage enzyme allowing up to 2 missing cleavages, 4 modifications per peptide and 5 charges. Mass error was set to 10ppm for precursor ions and 0.02Da for fragment ions. False discovery rate (FDR) thresholds for protein, peptide and modification site were specified at 1%. Minimum peptide length was set at 7. All the other parameters in MaxQuant were set to default values.

### GO and KEGG analysis

Gene Ontology (GO) annotation proteome was derived from the UniProt-GOA database (http://www.ebi.ac.uk/GOA/). Acetylation proteins were classified by Gene Ontology annotation based on three categories: biological process, cellular component and molecular function. Kyoto Encyclopedia of Genes and Genomes (KEGG) database was used to annotate protein pathway. Domain annotation was performed by using InterProScan on InterPro domain database via Web-based interfaces and services. WoLF PSORT was used for subcellular localization predication. The CORUM database was used to annotate protein complex.

### Functional enrichment analysis

Fisher's exact test was used to test for enrichment or depletion (right-tailed test) of specific annotation terms among members of resulting protein clusters. Derived *p* values weres further adjusted to address multiple hypotheses testing by the method proposed by Benjamini and Hochberg. Any terms having adjusted *p* values below 0.05 in any of the clusters were treated as significant.

### Enrichment-based clustering analysis

All the acetylation substrates categories obtained after enrichment were collated along with their *p* values, and then filtered for those categories which were at least enriched in one of the clusters with *p* value < 0.05. This filtered *p* value matrix was transformed by the function x = −log10 (*p* value). Finally these x values were z-transformed for each category. These z scores were then clustered by one-way hierarchical clustering (Euclidean distance, average linkage clustering) in Genesis. Cluster membership was visualized by a heat map using the “heatmap.2” function from the “gplots” R-package.

### Western blot

Cells were lysed in lysis buffer containing 150 mM NaCl, 1%NP40, 0.5%deoxycholic acid, 0.1%SDS, 50 mMTris (pH 8.0), and 1:25 protease inhibitor cocktail. Protein concentrations of the lysates were determined by the Bradford protein assay system (Bio-Rad, Hercules, CA). Equal amounts of protein (30 μg protein each lane) were separated by SDS-PAGE, transferred to nitrocellulose membranes (Hybond C, Amersham, UK). Immunoblots were blocked with 5% skim milk in TBS/Tween20 (0.05%, v/v) for 1 hour at RT. The membrane was incubated with primary antibody overnight at 4°C. Primary antibodies used include ING5 antibody (Proteintech Group, Inc. IL), p21 antibody (Santa Cruz Biotechnology Inc.), antibodies for Ac-H3K18, Ac-H4K16, p53, Ac-p53K382, Bax (Abcam), β-actin (Actin) antibody (Sigma), and pan-Acetyl antibody (Jingjie PTM Biolab). The membrane was incubated with corresponding secondary antibody conjugated with horseradish peroxidase (Sigma) (1:5000) at RT for 1h. The blots were developed using an enhanced chemiluminescence western blotting detection system (Amersham Bioscience, UK).

### Promoter assay

Luciferase assays for p53-responsive promoter activity were carried out as described [14] using the Dual-Luciferase reporter assay system (Promega,Madison,WI). A Renilla luciferase plasmid driven by a SV40 promoter and the luciferase reporter and expression construct were used to co-transfect A549 and HCT116 cells with expression vectors containing ING5. The promoter activity was determined as the ratio of firefly luciferase luminescence divided by Renilla luciferase.

### Statistical analysis

Data are shown as means plus SD from three independent experiments. Statistical comparisons were made using students’ *t*-test. *p* < 0.05 was considered statistically significant.

## SUPPLEMENTARY MATERIALS FIGURES AND TABLE




