# Supplementary material for: ING5 differentially regulates protein lysine acetylation and promotes p300 autoacetylation
Source: Oncotarget. 2017 Oct 31;9(2):1617–29. doi: 10.18632/oncotarget.22176 (PMC5788586; doi:10.18632/oncotarget.22176)
Supplement: Supplementary file 1 [file oncotarget-09-1617-s001.pdf]

ING5 differentially regulates protein lysine acetylation and promotes p300 autoacetylation

SUPPLEMENTARY MATERIALS

SUPPLEMENTARY DISCUSSION

ING5 overexpression upregulates acetylation of proteins with diverse functions

Histone acetylation catalyzed by histone acetyletransferase (HAT), is a reversible event that reduces chromosomal condensation to promote transcription. Histone deacetylase (HDAC) enzymes are co-repressors that induce gene silencing by deacetylating histones and subsequent chromosomal condensation. The HAT and HDAC enzymes could also target non-histone proteins. Thus, acetylation/deacetylation plays a more important role in cellular biology than simply transcriptional regulation.

Among all the ING5-accelerated acetylated proteins, the small heat shock protein family member heat shock protein beta-1 (HSPB1 or HSP27) rank the first with the highest fold (8.425) of acetylation at K123. As upregulation of HSP27 is associated with tumor growth and metastasis [30], together with what we have found that ING5 inhibits lung cancer invasiveness, whether ING5 promoted HSP27K123 acetylation has any effects on cancer metastasis deserves further investigation.

ING5 also promotes acetylation of proteins involved in DNA replication and DNA damage repair. Minichoromosome maintenance (MCM) proteins play essential roles as a replicative helicase in eukaryotic DNA replication during cell cycle progression [31]. These proteins are found to be overexpressed in cervical cancer cells [32]. Phosphorylation of the DNA replication licensing factor MCM4 at specific sites regulates the helicase activity of MCM4, 6, 7 complex [33]. ING5 overexpression increased acetylation of MCM4 at K413 (novel site) which may affect the helicase activity by crosstalking with phosphorylation and thus have effects on DNA replication. MSH6 belongs to DNA mismatch repair (MMR) proteins, whose acetylation is significantly promoted by ING5 overexpression at K504 by 7.12 fold, ranking the second highest acetyl-lysine site. DNA-dependent protein kinase (DNA-PK) complex, formed by the catalytic subunit *DNA-PK*cs (PRKDC) and the Ku70 (XRCC6)/Ku86 (XRCC5) regulatory heterodimer, is involved in DNA double-strand break (DSB) repair [34]. In the current study, we have found 18 acetyl-lysine sites in PRKDC with 15 novel sites, and 3 increased acetyl-lysine sites (K2928 by 1.61 fold, K3669 by 1.58 fold and K1985 by 1.46 fold). Ku80 acetylation at K660 is accelerated by 4.43 fold upon ING5 overexpression. Though 4 novel acetyl-lysine sites are found in Ku70, ING5 does not affect their acetylation level. The results may suggest a role of ING5 in regulation of DNA-replication and DNA damage repair through a post-translational modification mechanism.

Protein acetylation increases the functional diversity of a protein in stability, subcellular localization, enzymatic activity, protein-protein interaction, and crosstalk with other PTMs [4]. By regulating acetylation level of proteins involved in different cellular functions and processes, ING5 is expected to take part in a wide variety of roles through diverse mechanisms.

ING5 overexpression downregulated acetylation of proteins involved in metabolism

ING5 is a component of HAT enzyme complexes, which catalyze acetylation of histone and non-histone proteins. Unexpectedly, our data show that ING5 overexpression also promotes deacetylation of proteins, which are enriched in metabolic pathway, glycolysis/gluconeogenesis and HIF-1 signaling pathway, though the overall extent of ING5-promoted deacetylation is weaker than acetylation. The mechanisms of ING5-increased protein deacetylation might include upregulation of specific HDACs by ING5 or forming complexes with HDACs, which need further investigation.

Lysine acetylation plays an essential role in metabolism [2, 35–37]. Most enzymes involved in central metabolic pathways such as glycolysis, the TCA cycle,

carbon metabolism and glycogen metabolism, are acetylated in response to metabolic changes. Metabolism reprogramming characterized by increased cytoplasmic glycolysis and suppression of mitochondrial oxidative phosphorylation is a critical hallmark during cancer development known as "Warburg Effect" [38,39]. L-lactate dehydrogenase A chain (LDHA) and pyruvate dehydrogenase E1 component subunit alpha (PDHA1) are two important determinants between cytoplasmic glycolysis and mitochondrial oxidative phosphorylation in glucose metabolism [40, 41]. LDHA is a key enzyme for glycolysis in cytoplasm whose acetylation is downregulated by ING5 overexpression at K278 (novel site, 1.35 fold), K243 (1.36 fold), K5 (1.38 fold), K118 (1.44 fold) and K232 (1.56 fold). The acetylation of mitochondrial PDHA1 is also decreased by ING5 at K321 by 1.62 fold. PDHA1 deacetylation at K321 has been confirmed to increase its enzyme activity, leading to a reverse of "Warburg Effect" by switching from glycolysis to oxidative phosphorylation [42]. The association between ING5-induced inhibition of cancer invasion and PDHA1 acetylation is under further investigation.

The aldehyde dehydrogenases (ALDH) catalyze the conversion of acetaldehyde into acetic acid. There are two major isoforms of ALDH including ALDH1A1 and ALDH2, which locates in cytosol and motichondria, respectively. The enzymatic activity of human ALDH has now been employed as a cancer stem cells (CSCs) marker in various tumors [43–45]. The current data show that ING5 overexpression downregulated acetylation of both isoforms of ALDH. We have found 13 lysine acetylation sites in ALDH1 with 7 novel sites, and acetylation is decreased by ING5 at 5 lysine sites including K410 (1.31 fold), K495 (1.39 fold), K22 (1.43 fold), K139 (1.47 fold) and K128 (1.56 fold). K368 acetylation of ALDH2 is also downregulated by ING5 by 2.82 fold. These data may suggest a regulatory effect of ING5 on ALDH enzymes activity.

In addition, the cytoplasmic nicotinamide N-methyltransferase (NNMT) acetylation is downregulated by ING5 overexpression at K26 (2.49 fold), K47 (2.78 fold), K43 (2.93 fold) and K39 (3.38 fold), among which K26, K47 and K43 are novel acetyl sites. NNMT catalyzes the N-methylation of nicotinamide and other pyridines to form pyridinium ions. NNMT has been reported to change epigenetic states of cancer cells to hypomethylated histones with increased expression of pro-tumorigenic gene products by consuming methyl units [46]. Thus, whether ING5-downregulated acetylation of NNMT influences its enzyme activity and its significance deserve further investigation.

The mitochondrial creatine kinase U-type (CKMT1) K58 in the Phosphagen kinase domain is the most downregulated novel acetylation site (4.98 fold) by ING5 overexpression in the current study. CKMT1 plays a central role in energy transduction by reversibly catalyzing the transfer of phosphate between ATP and various phosphogens. CKMT1 is a key regulator of mitochondrial permeability transition pore whose depletion induces mitochondrial depolarization and apoptotic cell death [47]. The expression of CKMT1 and NCOA1 has recently been identified to have prognostic significance in advanced-stage head and neck carcinoma [48]. It would be interesting to define how lysine acetylation regulated by ING5 influence the activity of the CKMT1 enzyme and subsequently cellular biological processes.

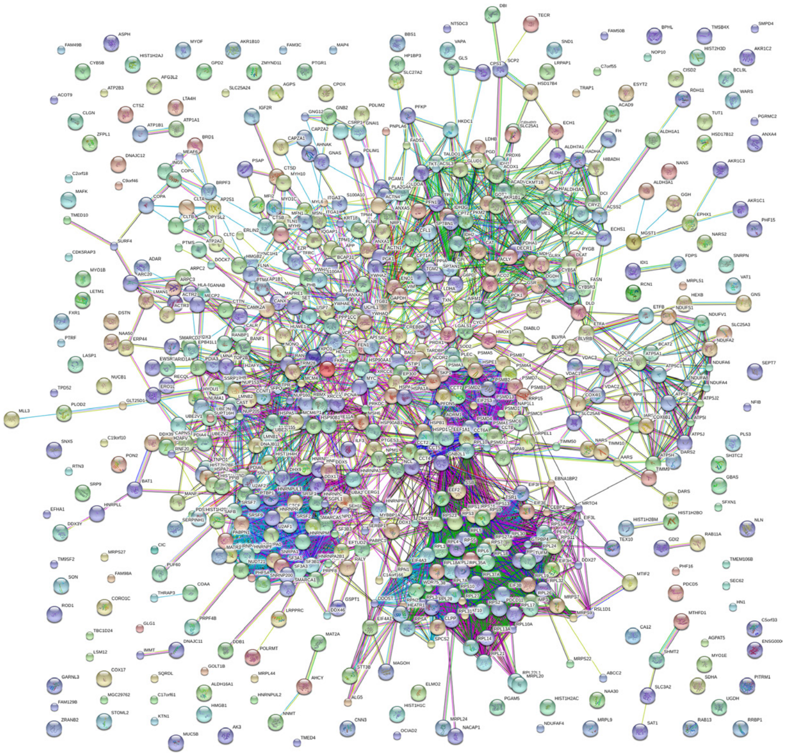

Supplementary Figure 1: Analysis of Interaction Network in lysine acetylome of A549 cells. Protein-protein interaction network on the basis of the STRING database.

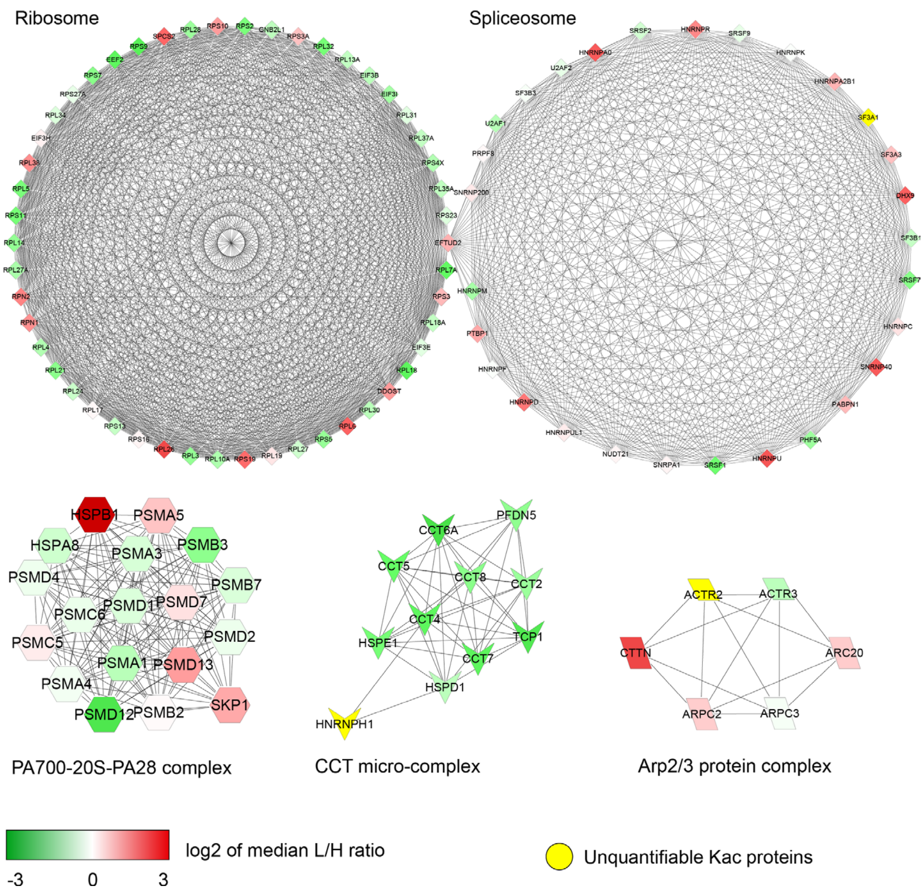

Supplementary Figure 2: Protein complex analysis of lysine acetylome of A549 cells.

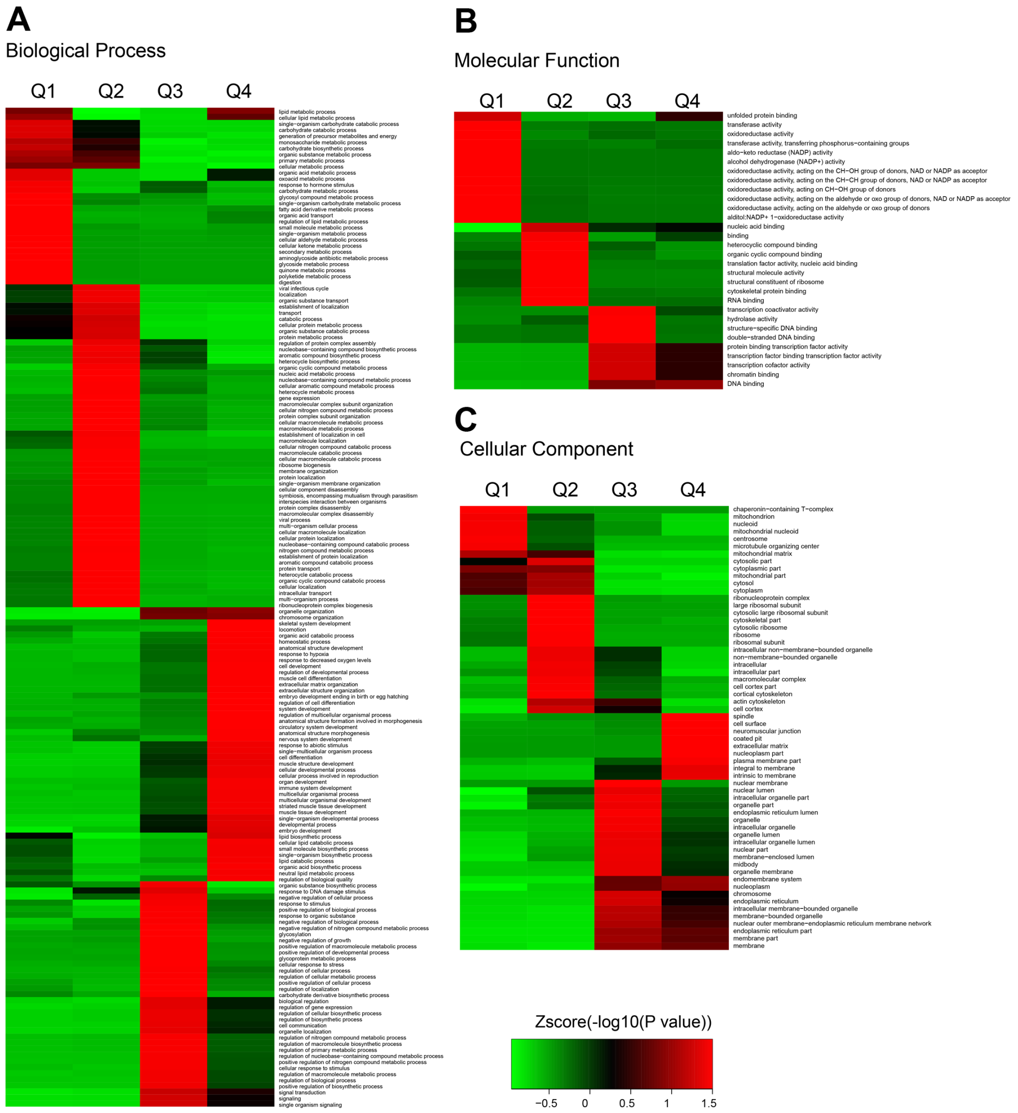

Supplementary Figure 3: Enrichment and clustering analysis of Lys acetylation proteome regulated by ING5 based on Gene Ontology annotation. (**A**) Biological process. (**B**) Molecular function. (**C**) Cellular component.

Supplementary Table 1: List of all acetylation sites identified from A549 control and A549 ING5 cells. See Supplementary_Table_1
